# Combined serum free light chain predicts prognosis in acute kidney injury following cardiovascular surgery

**DOI:** 10.1080/0886022X.2021.2013886

**Published:** 2022-01-27

**Authors:** Wenji Wang, Lulu Zhang, Tianye Yang, Shaojun Ma, Qi Zhang, Peng Shi, Feng Ding

**Affiliations:** a School of Medicine, Division of Nephrology, Shanghai Ninth People’s Hospital, Shanghai Jiaotong University, Shanghai, PR China; b Department of Medical Statistics, Children’s Hospital; Center for Evidence-based Medicine, Fudan University, Shanghai, PR China

**Keywords:** Acute kidney injury, free light chain, prognosis, all-cause mortality

## Abstract

**Objectives:**

Increased polyclonal free light chains (FLCs) are found in inflammatory conditions. Inflammation is recognized in the progression of acute kidney injury (AKI). This study was aimed to determine whether polyclonal combined FLC (cFLC) was associated with prognosis of AKI patients.

**Methods:**

This prospective cohort included 145 adults with hospital-acquired AKI following cardiovascular surgery between 2014 and 2016, according to the KDIGO creatinine criteria. The primary end point of the study was all-cause death during follow-up.

**Results:**

The median of serum cFLC concentration in the cohort was 42.0 (31.9–60.3 mg/L) and levels of cFLC in patients with AKI stage 3 were higher than those in AKI stage 1 and stage 2. cFLC levels correlated significantly with renal function biomarkers, high sensitivity C-reactive protein (hsCRP), and sequential organ failure assessment (SOFA) score. Patients were organized into the following two groups: the low-cFLC group (cFLC <43.3 mg/L) and the high-cFLC group (cFLC ≥ 43.3 mg/L). A total of 17 (11.0%) patient deaths occurred within 90 d, 13 (18.8%) in the high-cFLC group. Kaplan–Meier analysis revealed that the two groups differed significantly with respect to 90-d survival (log-rank *p* = .012), and Cox regression analysis showed that an cFLC level ≥43.3 mg/L was significantly associated with a 5.0-fold increased risk of death (adjusted hazard ratio [HR], 5.95; 95% confidence interval [CI], 1.04– 33.91; *p* = .045) compared with an cFLC level <43.3 mg/L.

**Conclusions:**

Serum cFLC levels were significantly elevated and might be an independent predictor of mortality in patients with AKI following cardiovascular surgery.

## Introduction

Acute kidney injury (AKI) is one of the most severe complications of cardiac surgery and is associated with increased mortality, morbidity, prolonged hospital stay, and progression to chronic kidney disease (CKD) even end stage kidney disease (ESKD) [1–3]. The incidence of AKI after cardiac surgery is higher than other surgeries and in general individuals [2]. Despite advances in AKI diagnosis and treatment, the mortality caused by this condition remains high [4,5]. It is important to distinguish severe AKI early and exactly after cardiac surgery. Some factors have traditionally served as mortality risk factors in AKI, and systemic inflammation is believed to play an important role in the pathogenesis and prognosis of AKI [6].

The immunoglobulin light chains, as part of the immunoglobulins, are produced by plasma cells. There are two isotypes of light chains, kappa (κ) and lambda (λ) [7]. Monoclonal production of free light chains (FLCs) is important in almost all plasma cell disorders, such as multiple myeloma, amyloidosis, and monoclonal gammopathy of undetermined significance [8]. Recently, elevated concentrations of serum polyclonal combined FLCs (cFLC), as a potential biomarker of activation of the B-lymphocyte, have been investigated in a number of non-hematological malignant and inflammatory disorders including diabetes, CKD, and systemic lupus erythematosus (SLE) [9–11]. Nonspecific increases in cFLC occurred in a similar to the nonspecific increases in C reactive protein seen with inflammation[12]. The elevation of cFLC is also the result of reduced removal by impaired renal function [7,13]. Furthermore, elevated cFLC was demonstrated as a predictor of poorer overall survival in the general population, in patients with heart failure and in patients with CKD, as well [14–16].

However, the data on concentrations of serum cFLC in AKI are limited and the relationship between its concentration and the life expectancy in patients with AKI remains unknown. Thus, we prospectively observed concentrations of serum cFLC and assessed the utility of serum cFLC as an independent predictor of mortality in patients with AKI following cardiovascular surgery.

## Patients and methods

### Study design

This study adhered to the Declaration of Helsinki and was approved by the ethics committee of Shanghai Ninth People’s Hospital, School of Medicine, Shanghai Jiaotong University (approval number: [2014]45). All experimental protocols were performed in accordance with the relevant guidelines and regulations. Written informed consent was obtained from either the patients or their legal guardians prior to their participating in the study.

**Figure 1.**
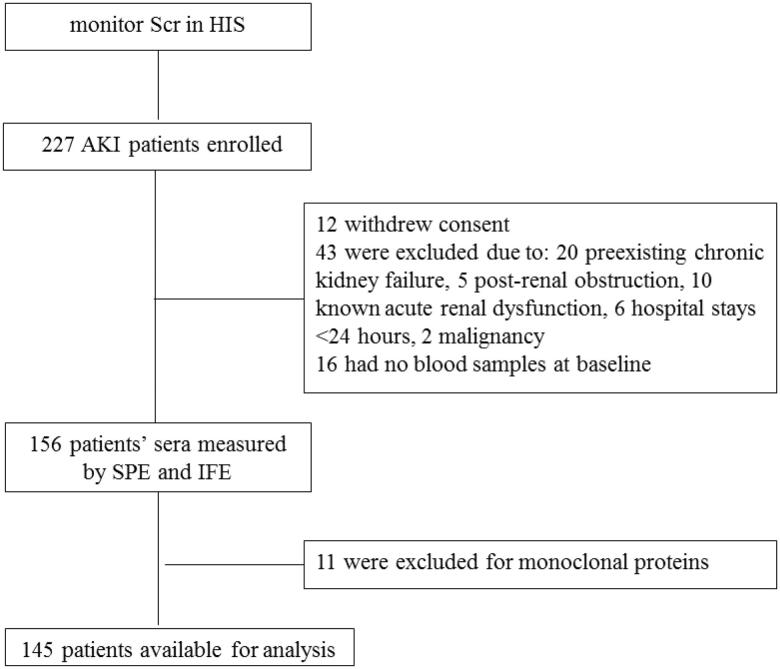
Flow chart of study progress. Scr: serum creatinine; HIS: hospital information system; SPE: serum protein electrophoresis; IFE” immunofixation electrophoresis.

Of the 227 patients enrolled in the study, 43 patients were excluded, 12 patients withdrew consent, and 16 patients provided no blood samples at baseline. Then 11 patients recruited in the cohort were excluded for their monoclonal FLCs which were indicated by serum protein electrophoresis (SPE) and immunofixation electrophoresis (IFE). Consequently, 145 patients were followed prospectively from the day of AKI diagnosed. The observational period of the study was 90 d. The primary outcome was all-cause mortality and the secondary endpoints were the need for renal replacement therapy (RRT) and recovery of renal function at hospital discharge defined as serum creatinine at discharge return to less than 50% change from baseline creatinine (Figure 1).

### Study population and definitions

Consecutive patients aged ≥18 years with the development of incident AKI following cardiac surgery and complete data were prospectively entered into the cohort from July 2014 to January 2016. Patients with preexisting CKD stages 3–5 (defined as an estimated glomerular filtration rate [eGFR] < 60 mL/min per 1.73 m^2^), with post-renal obstruction or rapidly progressive glomerulonephritis as the main cause of AKI, with known acute renal dysfunction before operations, with hospital stays <24 h, or with malignancy were excluded. AKI was determined using the 2012 Kidney Disease: Improving Global Outcomes (KDIGO) creatinine criteria [17]. Baseline eGFR was calculated by applying the Modification of Diet in Renal Disease (MDRD) equation, with appropriate adjustments for female patients [18].

### Data collection and sample handling

The serum creatinine levels of the inpatients, who underwent cardiac surgery, were monitored daily by the hospital information system (HIS). Patients with creatinine levels that rose as specified by the KDIGO criteria within 1 week were evaluated by nephrologists within 24 h. Data were collected daily by the nephrologists, and all of the collected data were extracted from electronic medical records. For each patient, the investigators entered the data into a computer form using EpiData software. The following information was recorded: ID number, demographic characteristics (age, gender, comorbid conditions, medical history, and operative method), illness severity (evaluated on the day of AKI diagnosis using the sequential organ failure assessment [SOFA] score), and renal functional parameters (serum creatinine, urea nitrogen, and AKI stage). Information pertaining to biochemical parameters (at the time of AKI diagnosis), including serum albumin, calcium, phosphate, intact parathyroid hormone (iPTH), high sensitivity C-reactive protein (hsCRP), hemoglobin, neutrophils counts, and hemoglobin, was also recorded. Blood samples were collected at the time of AKI diagnosis. A portion of each biological sample was measured immediately in the hospital laboratory, and the remainder of the sample was frozen and stored at −80 °C for subsequent quantification of FLCs and neuropeptide Y (NPY) levels.

### Biochemical measurements

Most biochemical parameters were measured in the hospital clinical laboratory with automated methods. The concentration of hsCRP was measured using immunoturbidimetry assay. Serum samples were also measured for FLC κ and FLC λ (Freelite, The Binding Site Group Ltd, Birmingham, UK) and NPY (Abbkine Scientific Co. Ltd, California, USA) by ELISA kits. Assays were run in accordance with the manufacturer’s instructions. All of the results were expressed in milligrams per liter. The cFLC level was calculated for each sample by addition of the FLC κ and FLC λ values. The published percentile normal range for serum cFLC is 9.3–43.3 mg/L [14].

### Statistical analyses

This analytical plan followed by the STROBE recommendations for observational cohort studies.

Categorical variables were expressed as frequencies (percentage), and continuous variables were expressed as mean ± SDs or as medians with interquartile ranges. Comparisons of categorical and continuous data for patients in the *a priori*-selected groups were performed by chi-squared tests and independent samples *t* tests or the Kruskal–Wallis test, respectively, as appropriate.

Spearman correlations were undertaken to analyze the correlation between cFLC levels and other variables. Kaplan–Meier analyses were used to assess the differences in surviving proportions between the high cFLC group and the low cFLC group. Receiver-operating characteristic (ROC) curves and areas under the curves (AUCs) were used to compare the predictability of the FLC levels for the 90-d mortality. To calculate the relative risks of all-cause death, hazard ratios (HRs) were obtained using Cox proportional hazard models after controlling for confounding variables. Univariate Cox regression was performed to identify potential confounding variables, including comorbidities (diabetes mellitus, coronary heart disease, and hypertension), surgery method, SOFA scores, and biochemical parameters. The multivariable Cox regression model consisted of variables with a *p-*value <0.1 in the univariate Cox regression model or clinical value.

All *p-*values were two tailed and *p-*values <.05 were considered statistically significant. Statistical analyses were performed with SPSS version 19.0 (SPSS, Chicago, IL) and SAS version 9.0 (SAS Inc., Cary, NC).

## Results

### Study cohort

Of the 227 patients enrolled in the study, 43 patients were excluded, 12 patients withdrew consent, and 16 patients had no serum samples at baseline. Then 11 patients were excluded for monoclonal proteins as indicated by SPE and confirmed by IFE. Consequently, 145 patients were followed for 90 d prospectively (Figure 1).

### Serum free light chains in patients with AKI

**Figure 2.**
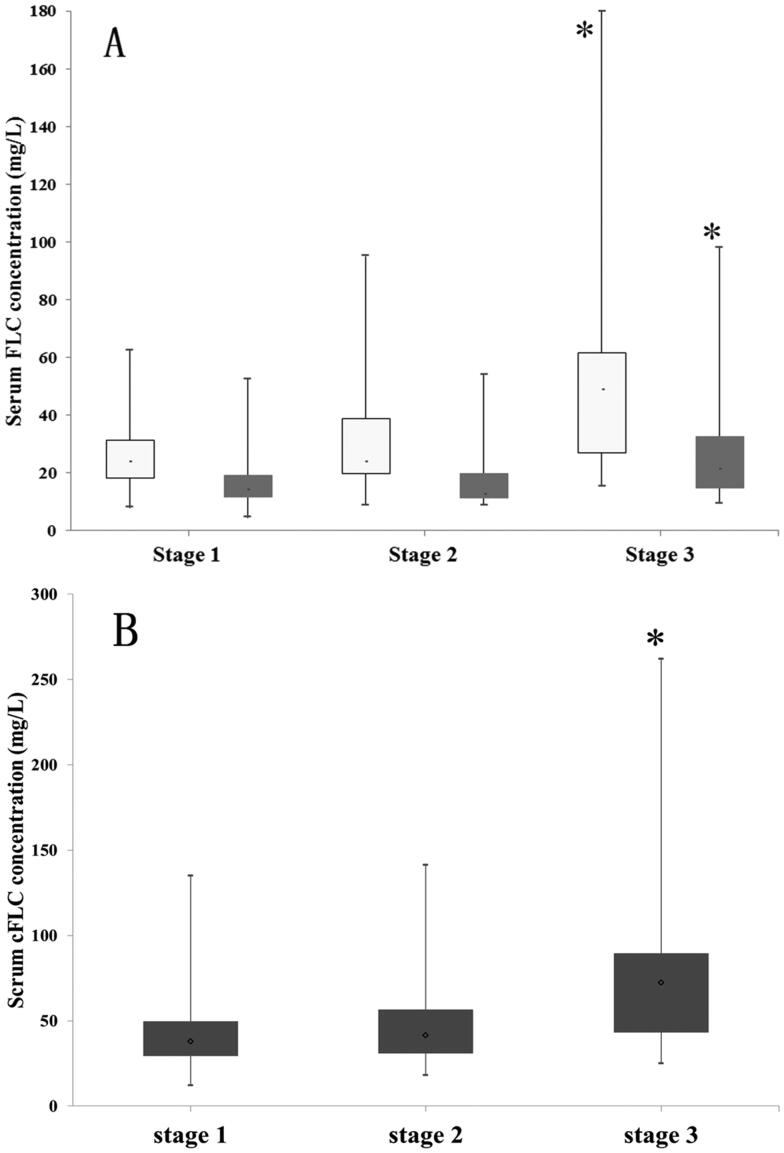
Serum free light chain concentrations in patients with different AKI stages. (A) Serum free light chain (FLC) κ (white blocks) and FLC λ (grey blocks), (B) combined free light chain (cFLC) concentrations increased in patients with AKI stage 3 *versus* patients with stages 1 and 2 (*p<.001). Median and ranges are indicated (black bars).

The median concentration of serum FLC κ in this AKI cohort was 26.8 mg/L (20.1–41.6 mg/L) and serum FLC λ was 14.8 mg/L (11.9–20.6 mg/L) at the time of AKI diagnosis. The median level of cFLC was 42.0 mg/L (31.9–60.3 mg/L). As shown in Figure 2(A), both FLC κ and FLC λ concentrations in patients with AKI stage 3 were significantly higher than those in patients with AKI stages 1 and 2: FLC κ 49 mg/L (27–61.7 mg/L) *versus* 24 mg/L (18.4–31.3 mg/L) and 27 mg/L (19.9–38.7 mg/L), and FLC λ 21.4 mg/L (14.7–32.5 mg/L) *versus* 14.1 mg/L (11.8–19.1 mg/L) and 12.8 mg/L (11.3–19.8 mg/L). Patients with AKI stage 3 also had significantly raised serum concentrations of cFLC compared to those with AKI stages 1 and 2 (72.45 mg/L *versus* 37.8 mg/L and 41.6 mg/L, respectively, both *p* < .001; Figure 2(B)).

Serum FLC concentrations were measured in 65 patients with CKD. The median concentration of cFLC in 145 patients with AKI were significantly lower than that in patients with CKD (42.0 mg/L *versus* 92.7 mg/L, *p* < .001), but higher than previous reported values in normal population [19] (median, 20.2 mg/L; Supplemental Figure 1).

### Clinical and biochemical characteristics of the AKI cohort

The cohort consisted of 102 men, with a mean age of 56.0 ± 12.7 years. The demographics, clinical features, and laboratory parameters at the time of AKI diagnosis are shown in Table 1. Baseline laboratory characteristics before cardiac surgeries were shown in Supplementary Table 1 and Supplementary Figure 2. Patients were categorized into the following two groups according to their serum cFLC concentrations, measured at the time of AKI diagnosis: a low-cFLC group (cFLC < 43.3 mg/L) and a high-cFLC group (cFLC ≥ 43.3 mg/L), based on the published 95th percentile normal range for cFLC is 9.3–43.3 mg/L [14]. cFLC levels ≥ 43.3 mg/L were observed in 69 (47.6%) patients. Patients in the high-cFLC group had higher SOFA score, serum creatinine, urea nitrogen, and hsCRP levels. Patients who underwent aorta operations had higher levels of serum cFLC.

**Table 1.** Demographics, clinical and laboratory characteristics in total serum cFLC and according to the 2 *a priori*-selected groups of serum cFLC levels in 145 patients.

	Total	cFLC < 43.3 mg/L	cFLC ≥ 43.3 mg/L	
	(*N* = 145)	(*N* = 76)	(*N* = 69)	*p* Value
Male, *n* (%)	102 (70.3%)	51 (67.1%)	51 (73.9%)	.370
Age, years	56.0 ± 12.7	55.0 ± 12.7	57.1 ± 12.7	.330
Comorbidities, *n* (%)				
Hypertension	72 (49.7%)	36 (47.4%)	36 (52.2%)	.563
Heart failure	48 (33.1%)	28 (36.8%)	20 (29.0%)	.315
Diabetes	18 (12.4%)	6 (7.9%)	12 (17.4%)	.083
Cerebrovascular disease	9 (6.2%)	4 (5.3%)	5 (7.2%)	.621
Operative method, *n* (%)	–	–	–	.161
Valve	78 (53.8%)	46 (60.5%)	32 (46.4%)	–
CABG	18 (12.4%)	11 (14.5%)	7 (10.1%)	–
Aorta	24 (16.6%)	8 (10.5%)	16 (23.2%)	–
Combined	11 (7.6%)	4 (5.3%)	7 (10.1%)	–
Others	14 (9.7%)	7 (9.2%)	7 (10.1%)	–
SOFA score	7.69 ± 4.11	6.68 ± 3.54	8.80 ± 4.42	.002
Cause of AKI, *n* (%)				.127
IR	67 (46.2%)	30 (39.5%)	37 (53.6%)	.088
Nephrotoxic drugs	10 (6.9%)	8 (10.5%)	2 (2.9%)	.070
Sepsis	7 (4.8%)	5 (6.6%)	2 (2.9%)	.302
Combined	61 (42.1%)	33 (43.4%)	28 (40.6%)	.729
RRT	26 (17.9%)	6 (7.9%)	20 (29.0%)	.002
Laboratory data				
FLC κ, mg/L	26.8 (20.1, 41.6)	20.3 (16.0, 23.7)	43.1 (32.7, 56.5)	<.001
FLC λ, mg/L	14.8 (11.9, 20.6)	12.1 (10.6, 13.4)	20.1 (18.1, 28.4)	<.001
cFLC, mg/L	42.0 (31.9, 60.3)	32.7 (27.8, 37.0)	52.8 (50.5, 85.2)	<.001
κ/λ ratio	1.74 (1.43, 2.18)	1.61 (1.33, 1.92)	1.97 (1.57, 2.40)	<.001
NPY, pg/mL	11.03 (7.03, 20.70)	11.03 (7.03, 21.70)	13.03 (7.03, 17.03)	.896
iPTH, pg/mL	127.5 (88.0, 183.2)	122.0 (90.8, 165.6)	130.3 (59.3, 202.1)	.620
Creatinine, μmol/L	150.5 (123.0, 187.8))	129.0 (115.0, 171.0)	174.0 (146.0, 217.0)	<.001
Urea nitrogen, mmol/L	14.2 ± 17.6	11.0 ± 3.5	17.7 ± 25.4	.027
Calcium, mmol/L	2.09 ± 0.22	2.09 ± 0.20	2.09 ± 0.23	.905
Phosphate, mmol/L	1.29 ± 0.57	1.28 ± 0.46	1.29 ± 0.66	.941
Albumin, g/L	35.1 ± 4.2	35.5 ± 4.8	34.6 ± 3.3	.226
hsCRP, mg/L(A)	1.50 (0.70, 4.97)	1.15 (0.53, 3.58)	1.90 (1.00, 9.62)	.032
Neutrophilic granulocyte, ×10^9^ cell/L	11.9 (9.2, 14.6)	11.85 (9.20, 14.20)	12.2 (9.13, 15.05)	.952
Hemoglobin, g/L	102.8 ± 17.2	105.1 ± 17.1	100.1 ± 17.0	.087

cFLC: combined free light chain; AKI: acute kidney injury; CABG: coronary artery bypass graft; SOFA score: sequential organ failure assessment score; IR: ischemia reperfusion; RRT: renal replacement therapy; FLC κ: κ free light chain; FLC λ: λ free light chain; κ/λ ratio: κ to λ free light chain ratio; NPY: neuropeptide Y; iPTH: intact parathyroid hormone; hsCRP: high sensitivity C-reactive protein.

Table 2 shows that cFLC correlated significantly with renal function biomarkers, serum creatinine, and urea nitrogen (rho = 0.485 and 0.559, respectively, *p* < .001). Weak but significant correlations were observed between cFLC and hsCRP and SOFA score (rho = 0.230 and 0.288, *p* = .012 and *p* < .001, respectively). cFLC correlated inversely with hemoglobin (rho= −0.248, *p* = .003).

**Table 2.** Spearman correlation of serum cFLC with other clinical parameters in AKI.

Clinical parameter	Spearman rho	*p* Value
Age	0.041	.627
SBP	0.043	.643
SOFA score	0.288**	<.001
Serum creatinine	0.485**	<.001
Urea nitrogen	0.559**	<.001
NPY	0.060	.660
iPTH	0.105	.309
Calcium	−0.058	.534
Phosphate	0.080	.393
Albumin	−0.118	.165
hsCRP	0.230*	.012
Neutrophilc granulocyte	0.029	.737
Hemoglobin	−0.248**	.003

cFLC: combined free light chain; AKI: acute kidney injury; SOFA score: sequential organ failure assessment score; NPY: neuropeptide Y; iPTH: intact parathyroid hormone; hsCRP: high sensitivity C-reactive protein;. *p<.05; **p<.01.

### Survival analysis

The overall in-hospital mortality was 9%. In the 90 d of follow-up, there were 17/145 (11.0%) deaths from all causes. The median cFLC level at the time of AKI diagnosed was significantly higher in patients who died compared with those who were alive at the end of the follow-up (73.7 mg/L *versus* 40.3 mg/L, *p*=.004). A total 13 of 17 (76.5%) deceased patients had elevated cFLC (≥43.3 mg/L).

**Figure 3.**
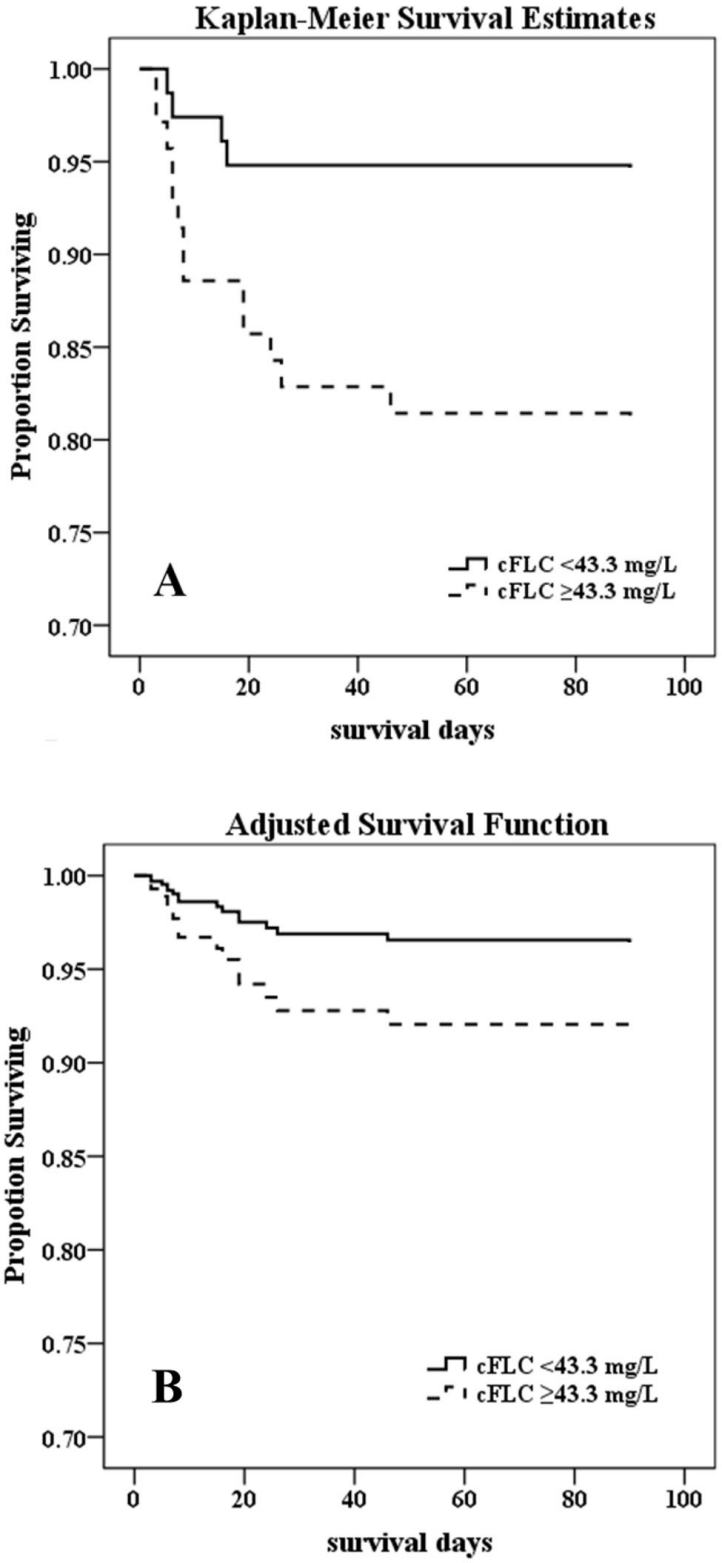
Kaplan–Meier proportion of surviving patients after 90 d of observation according to the groups of cFLC in 145 patients occurring AKI. (A) unadjusted, Log rank *p*=.012; (B) adjusted for age, gender, aorta surgery, SOFA, serum albumin, creatinine, and hsCRP, Log rank *p*=.038.

In the study, cFLC was evaluated as a potential new ‘mortality risk factor.’ Kaplan–Meier survival analysis revealed that patients in the high-cFLC group had worse overall survival than those in the low-cFLC group, both in the unadjusted model (Log-rank *p* = .012) and in the full-adjusted model (Log-rank *p* = .038) (Figure 3).

**Figure 4.**
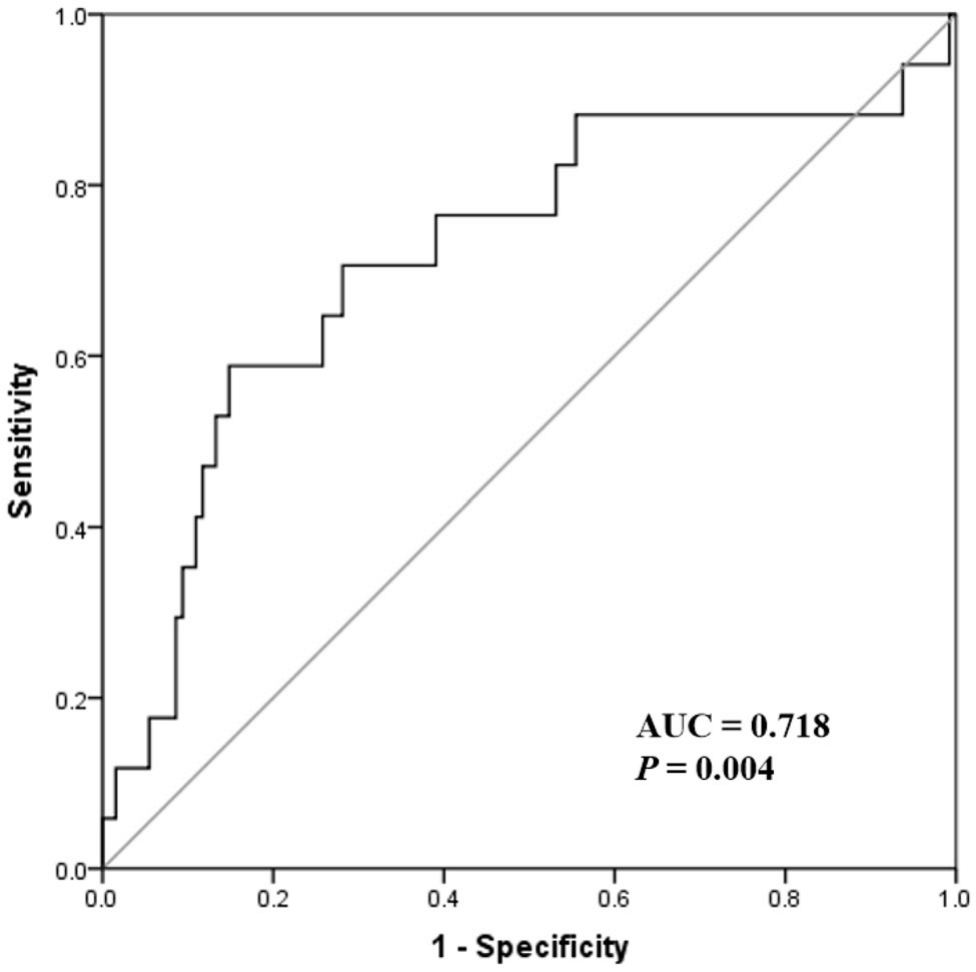
Receiver operating characteristic curve for the 90-d mortality risk according to the levels of combined serum free light chain.

Univariate Cox proportional hazards analysis identified age, undergoing aorta operation, higher SOFA score, elevated cFLC, creatinine, hsCRP, and decreased albumin as risk factors that were associated with all-cause 90-d mortality (Supplemental Table 2). Additionally, the AUC for the 90-d mortality risk according to the levels of serum cFLC was shown in Figure 4. The strength of the AUC for cFLC was 0.718 (95% CI, 0.572–0.865; *p* = .004) and the C-index was 0.707.

Patients with higher cFLC had an increased risk of death than patients with lower cFLC when serum cFLC was entered either as a dichotomous (HR, 5.951; 95% CI, 1.0449–33.911; *p* = .045) or as a continuous (HR, 1.012; 95% CI, 1.001–1.024; *p* = .046) variable, as demonstrated by Cox regression after adjusted multivariate analysis (Table 3).

**Table 3.** Multivariate Cox proportional hazard model of mortality during 90-d follow-up in AKI patients (cFLC entered as a continuous or dichotomous variable).

Model	HR	95% CI	*p* Value
cFLC (continuous variable)			
Unadjusted	1.010	1.003–1.018	.004
Model 1	1.009	1.003–1.016	.006
Model 2	1.012	1.001–1.024	.046
cFLC (dichotomous variable)			
Unadjusted	3.800	1.239–11.654	.020
Model 1	4.412	1.423–13.684	.010
Model 2	5.951	1.044–33.911	.045

Variables of model 1 include age and gender.

Variables of model 2 include age, gender, aorta surgery, SOFA score, serum albumin, creatinine, and hsCRP.

### Secondary endpoints

There were 6 of 76 patients in the low-cFLC group and 20 of 69 patients in the high-cFLC group underwent dialysis. The difference between the two groups was significant (7.9% *versus* 29%, *p*  = .01). The serum creatinine at hospital discharge was available in 73 of 76 patients in the low-cFLC group and 65 of 69 patients in the high-cFLC group. The number of patients with recovered renal function in the low-cFLC group was more than that in the high-cFLC group (91.8% *versus* 64.4%, *p* < .001). Elevated serum cFLC at the time of AKI diagnosis was adverse to recovery of renal function at discharge both in univariate Log regression model (OR, 0.983; 95% CI, 0.971–0.995; *p* = .005) and in multivariate model adjusted by age and gender (OR, 0.982; 95% CI, 0.970–0.995; *p* = .005).

## Discussion

In this prospective study, serum cFLC concentrations were increased in almost one-half of patients with AKI following cardiovascular surgery. Elevated cFLC was significantly correlated with renal function markers, hsCRP, and SOFA score. Of note, patients with high serum cFLC concentrations were at increased risk of all-cause death. Furthermore, the fully adjusted multivariable model showed that higher serum cFLC concentration remained an independent predictor of mortality with a 5-fold higher risk of death within 90 d after AKI diagnosis.

Polyclonal FLCs are products of intact immunoglobulin synthesis by plasma cells. Serum concentrations of FLCs are increased as the result of their excessive production when adaptive immune system was activated. For the serum half-lives of FLCs are 2–6 h [20], polyclonal FLC may be a rapid responder to inflammation. Elevated polyclonal cFLC has been reported in autoimmune and other chronic inflammatory conditions, such as SLE, rheumatoid arthritis, heart failure, and diabetes [9–11,15]. Jackson CE et al. reported that 43% patients had elevated cFLC concentrations (>45.7 mg/L) at the baseline in a cohort of 628 patients with heart failure [15]. Besides, cFLC levels showed a relatively small but significant increase in patients with acute STEMI with peak concentrations of 28 mg/L *versus* 24 mg/L at the baseline [21]. On the other hand, because of the elimination of FLCs from the serum by glomerular filtration, impaired renal function also results in elevation of serum cFLC concentrations [22]. In the previous studies, the median serum cFLC concentration was 36.3 mg/L in patients with CKD stage 3 and was 210 mg/L in patients with CKD stage 5 on hemodialysis [16,23]. In this AKI cohort, the median level of serum cFLC was 42 mg/L which higher than previous reported values in normal population [19] but lower than patients with CKD stages 3–5 without dialysis. The study also showed that patients with AKI stage 3 had highest levels of serum cFLC and there was a significant correlation between cFLC and markers of kidney function, including serum creatinine and urea nitrogen in patients with AKI. These findings are similar with the results of Hutchison’s study which showed a progressive increase in serum FLCs concentrations with CKD stage and strong correlations of FLCs with several biomarkers of renal function [11]. Thus, the elevated levels of serum cFLC in AKI may be the result of both immune system activation and reduced glomerular filtration.

In the last decade, studies have revealed a relationship between elevated polyclonal cFLC levels and poor outcomes in different populations. In autoimmune diseases, such as rheumatoid arthritis and Sjogren’s syndrome, increased concentrations of serum FLCs were associated with disease activity [24]. And circulating serum cFLC concentrations were associated with an increased risk of 76% for new percutaneous coronary intervention in patients with acute coronary syndrome [21]. Moreover, in two large cohorts, elevated cFLC levels were predictive for worse overall survival [14,25]. Dispenzieri et al. reported that for each higher decile of cFLC, there was an increased risk of mortality in 15,859 people without plasma cell disorders from Olmsted County, Minnesota [14]. Similarly, Anandram et al. showed that elevated cFLC was an independent risk factor for death in patients who had FLC measured to screen for a monoclonal disease but had no evidence of monoclonal disease after 4.5 years follow-up [25]. Another study has reported that higher cFLC concentration was associated with reduced survival independent of hsCRP in patients with CKD stage 3 recently [16]. However, there is little data on cFLC and its relationship with outcomes in AKI. Patients with AKI following cardiovascular surgery were included in this study. As expected, there was a significantly increased risk of death within 90 d after the diagnosis of AKI in this cohort. Higher concentrations of serum cFLC had increasing HR and patients in the group with cFLC ≥ 43.3 mg/L had the largest HR of 5.95 associated with all-cause mortality even in multivariate analysis, with an abnormal cFLC in 76.5% deceased patients.

The underlying roles of elevated cFLCs in the prognosis of AKI are uncertain. There is evidence that cFLC levels are independently associated with extend of carotid atherosclerosis and CVD risk in patients with diabetes [26]. Furthermore, an inverse linear relationship between levels of serum FLCs and left ventricle function was found in acute ischemic heart failure in patients without kidney failure and a 2-fold risk of cardiovascular death was observed in the hospital referral population with higher serum cFLC [25,27], which may suggest the poor outcome of patients, undergoing cardiovascular surgery in our study, with high cFLC levels. On the other hand, studies have shown that several markers of inflammation were associated with the development of AKI [28–30]. It is also possible that elevated cFLC is a nonspecific marker of immune system activation in AKI. Gudowska-Sawczuk and Mroczko reported that FLCs in serum and CSF could provide rapid information about intrathecal inflammation in patients with multiple sclerosis [31]. Studies demonstrated that FLCs participated in inflammatory conditions by activating mast cells [32] and by increasing infiltration of active neutrophils [33]. Thus, serum cFLC elevation may be an immune response to releasing of endotoxins, which was a result of disruptions of gut barrier function in AKI [34]. And FLCs may contribute to poor outcomes of AKI by activating inflammation.

A number of systematic reviews and investigations demonstrated that some novel biomarkers may predict outcomes of AKI over the last decade. Cystatin C is commonly used as a measure for GFR and is associated with various immune responses [35,36]. It was shown that cystatin C was significantly higher among those patients with acute respiratory distress syndrome (ARDS) who died and subjects with AKI in the highest quartile of cystatin C had a significantly higher odds of death at 60 d compared to subjects in the lower cystatin C (OR 1.6) [37]. The highest quartile of cystatin C also increased 74% risk of mortality among ICU patients with AKI [38]. However, after adding adjustment by the RIFLE classification, the point estimates for cystatin C are lowered (HR 1.41, *p* > .05). Neutrophil gelatinase associated lipocalin (NGAL) is one of the most extensively studied biomarkers used for AKI [39]. Plasma NGAL > 151 ng/mL was associated with higher 90-d mortality in multivariate model (HR 2.0) in the cardiogenic shock population and was also able to stratify patients with AKI of the population into high or low mortality risk groups [40]. The urinary NGAL could also help to predict the AKI severity in relation to death in a meta-analysis of nine studies with a total of 1948 patients [41]. The odds ratio for in-hospital mortality based on increased urinary and serum NGAL levels were 8.8. However, biomarkers including urine NGAL and urine tissue inhibitor of metalloproteinase-2 (TIMP-2) didn’t predict the mortality among decompensated cirrhotic patients with AKI and patients with community-acquired AKI [42,43]. In another meta-analysis of studies that used the urinary liver-type fatty acid-binding protein (L-FABP) to predict the in-hospital mortality, including three studies with 561 patients with AKI, the in-hospital mortality odds ratio was 13.7 [44]. In this study, the level of serum cFLC ≥ 43.3 mg/L was associated with all-cause mortality in patients with AKI following cardiac surgery even in the fully adjusted model (HR 5.95).

## Limitations

The study population was focused on patients undergoing cardiovascular surgery from a single center, which was a tertiary teaching hospital and the patients might be more complicated with other diseases. So the selection bias was inevitable and we used multivariate analysis to adjust for comorbidities and inflammatory markers. And patients with CKD stages 3–5 were excluded in the study. The findings may not be applicable to patients following other surgeries and patients with CKD stages 3–5 following cardiac surgery. Secondly, only a single measurement of cFLC concentration at the diagnosis of AKI was available for the patients. Thus, it is unknown whether the cFLC concentration changes over time or its change would be associated with prognosis of AKI. Thirdly, the other endpoints, such as end stage renal disease and cause of death, were not available. More outcome events are required to identify whether serum cFLC adds significantly to the power of previous described risk factors to predict poor outcomes in patients with AKI.

## Conclusion

Serum cFLC levels were found elevated in patients with AKI and a higher serum cFLC level was independently associated with a high risk of all-cause mortality in patients with in hospital-acquired AKI following cardiovascular surgery. This association remained after adjustment for confounding risk factors. These results may lead to the development of more useful strategies for identifying patients at high risk of death.

## Supplementary Material

Supplemental Material

## Data Availability

Data are available by contacting the corresponding author.

## Abbreviations

AKI: acute kidney injury
ARDS: acute respiratory distress syndrome
AUC: areas under the curve
cFLC: combined free light chain
CI: confidence interval
CKD: chronic kidney disease
eGFR: estimated glomerular filtration rate
ESKD: end stage kidney disease
FLC: free light chain
HIS: hospital information system
HR: hazard ratio
hsCRP: high sensitivity C- reactive protein
IFE: immunofixation electrophoresis
iPTH: intact parathyroid hormone
KDIGO: Kidney Disease: Improving Global Outcomes
L-FABP: liver-type fatty acid-binding protein
MDRD: modification of diet in renal disease
NGAL: neutrophil gelatinase associated lipocalin
NPY: neuropeptide Y
ROC: receiver-operating characteristic
RRT: renal replacement therapy
SD: standard deviation
SLE: systemic lupus erythematosus
SOFA: sequential organ failure assessment
SPE: serum protein electrophoresis
STROBE: STrengthening the Reporting of OBservational studies in Epidemiology
TIMP-2: tissue inhibitor of metalloproteinase-2
