# Supplementary material for: Combined serum free light chain predicts prognosis in acute kidney injury following cardiovascular surgery
Source: Ren Fail. 2022 Jan 27;44(1):1–10. doi: 10.1080/0886022X.2021.2013886 (PMC8797736; doi:10.1080/0886022X.2021.2013886)
Supplement: Supplemental Material [file IRNF_A_2013886_SM7513.pdf]

Combined serum free light chain predicts prognosis in acute kidney injury following cardiovascular surgery

Wenji Wang MD[1]; Lulu Zhang PhD[1]; Tianye Yang MD[1]; Shaojun Ma MD[1]; Qi Zhang MD[1]; Peng Shi MS[2]; Feng Ding MD PhD[1]

1. Division of Nephrology, Shanghai Ninth People's Hospital, School of Medicine, Shanghai Jiaotong University, Shanghai, China, 200011
2. Department of Medical Statistics, Children's Hospital; Center for Evidence-based Medicine, Fudan University, Shanghai, China, 200433

*** Corresponding author:**

Feng Ding MD, PhD

Division of Nephrology

Shanghai Ninth People's Hospital

School of Medicine

Shanghai Jiaotong University

639 Zhizaoju Road

Shanghai 200011, China

E-mail: dingfeng@sjtu.edu.cn

Tel: 86-21-53315165

Supplementary Table 1. Laboratory characteristics in in 145 patients before cardiac surgeries (baseline).

	Total (N=145)	cFLC <43.3mg/L (N=76)	cFLC ≥43.3mg/L (N=69)	*P* value
**Laboratory data**				
Creatinine, μmol/L	78 (67, 88)	79 (72, 88)	75 (66, 86)	0.085
Urea nitrogen, mmol/L	6.4 (5.1, 8.3)	6.2 (5.1, 7.5)	6.7 (5.2, 8.7)	0.150
Albumin, g/L	40 (38, 41)	40 (38, 42)	39 (37, 41)	0.054
hsCRP, mg/L	1.6 (0.8, 6.0)	1.3 (0.7, 4.2)	1.9 (1.0, 9.6)	0.159
Neutrophilic granulocyte, $\times 10^9$ cell/L	3.7 (2.8, 5.1)	3.5 (2.8, 4.5)	3.9 (3.2, 7.2)	0.054
Hemoglobin, g/L	134 (121, 148)	138 (123, 149)	132 (120, 144)	0.091

Supplemental Table 2. Univariate Cox proportional hazards analysis

Clinical Variables	*P*	HR	95% CI
Age	0.006	0.952	0.920-0.986
Gender	0.282	1.982	0.570-6.897
Hypertension	0.381	1.539	0.586-4.045
Heart failure	0.178	0.425	0.122-1.478
Diabetes	0.157	2.246	0.732,-6.890
Valvular operation	0.013	0.243	0.079-0.746
CABG	0.965	0.968	0.221-4.231
Aorta operation	0.001	5.316	2.048-13.802
Combined operation	0.555	1.559	0.356-6.818
Other operation	0.591	0.575	0.076-4.335
SOFA score	<0.001	1.262	1.116-1.427
FLC κ	0.001	1.018	1.007-1.028
FLC λ	0.055	1.018	1.000-1.037
cFLC	0.004	1.010	1.003-1.018
cFLC ≥ 43.3 mg/L	0.020	3.800	1.239-11.654
cFLC < 43.3 mg/L	reference	reference	reference
κ/λ ratio	0.136	1.856	0.824-4.184
NPY	0.496	1.012	0.977-1.049
iPTH	0.021	1.004	1.001-1.007
Creatinine	< 0.001	1.009	1.006-1.012
Urea nitrogen	0.727	1.004	0.984-1.024
Calcium	0.394	2.647	0.282-24.816
Phosphate	0.166	1.852	0.774-4.428
Albumin	0.002	0.831	0.741-0.932
hsCRP	0.007	1.022	1.006-1.038
Neutrophilic granulocyte	0.164	1.099	0.994-1.215
Hemoglobin	0.180	0.978	0.947-1.010

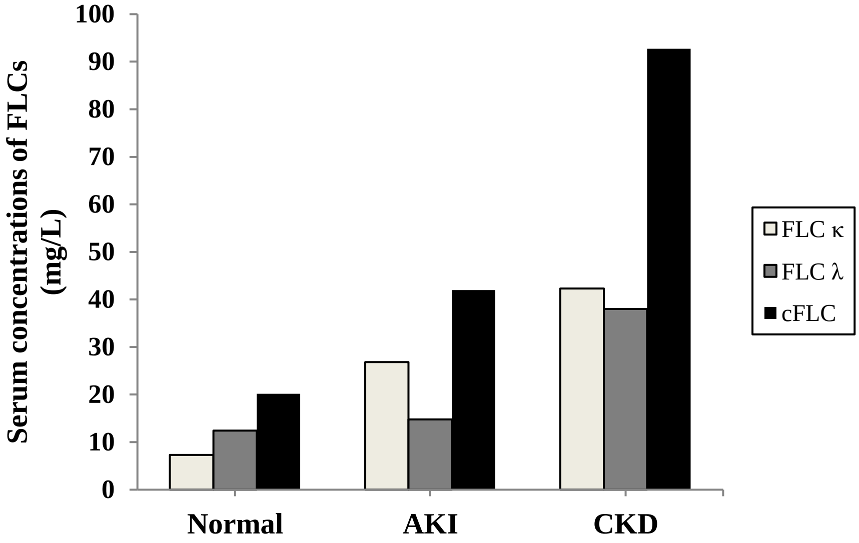

Supplemental Figure 1. Serum FLC concentrations in patients with AKI were higher in patients with acute kidney injury (AKI) compared to previously reported values in normal controls[Clin Chem 2002], but they were lower than values in patients with chronic kidney diseases (CKD).

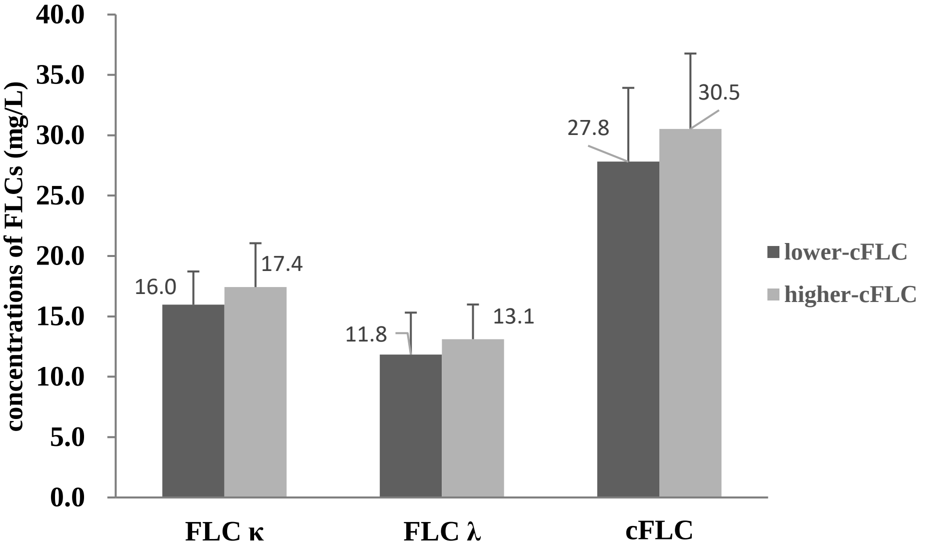

Supplemental Figure 2. Baseline concentrations of serum FLCs in 21 patients with serum samples before cardiac surgery. There was no difference between the lower-cFLC group (cFLC < 43.3 mg/L at the time of AKI diagnosis) and the higher-cFLC group (cFLC ≥ 43.3 mg/L at the time of AKI diagnosis), $P > 0.05$.
